# Dark-field X-ray microscopy for multiscale structural characterization

**DOI:** 10.1038/ncomms7098

**Published:** 2015-01-14

**Authors:** H. Simons, A. King, W. Ludwig, C. Detlefs, W. Pantleon, S. Schmidt, I. Snigireva, A. Snigirev, H. F. Poulsen

**Affiliations:** 1 Department of Physics, DTU, 2800 Kongens Lyngby, Denmark; 2 ESRF, CS 40220, 38043 Grenoble Cedex 9, France; 3 MATEIS, INSA-Lyon, 69621 Villeurbanne Cedex, France; 4 Department of Mechanical Engineering, DTU, 2800 Kongens Lyngby, Denmark; 5 Immanuel Kant Baltic Federal University, Kaliningrad 236041, Russian Federation

## Abstract

Many physical and mechanical properties of crystalline materials depend strongly on their internal structure, which is typically organized into grains and domains on several length scales. Here we present dark-field X-ray microscopy; a non-destructive microscopy technique for the three-dimensional mapping of orientations and stresses on lengths scales from 100 nm to 1 mm within embedded sampling volumes. The technique, which allows ‘zooming’ in and out in both direct and angular space, is demonstrated by an annealing study of plastically deformed aluminium. Facilitating the direct study of the interactions between crystalline elements is a key step towards the formulation and validation of multiscale models that account for the entire heterogeneity of a material. Furthermore, dark-field X-ray microscopy is well suited to applied topics, where the structural evolution of internal nanoscale elements (for example, positioned at interfaces) is crucial to the performance and lifetime of macro-scale devices and components thereof.

## Abstract (web summary)

The internal structure of materials determines many of their physical and mechanical properties. Here, the authors have developed a non-destructive X-ray microscopy technique for layer-by-layer mapping of crystallographic orientations and stresses to obtain a three-dimensional reconstruction of a material.

## Graphical abstract

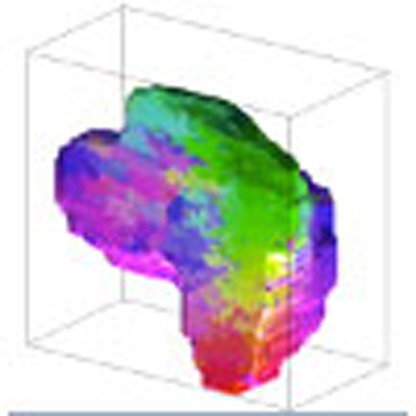

Crystalline materials are ubiquitous and include most metals, ceramics, semiconductors, rocks, ice, sand, bones and many artefacts of artistic and archaeological interest. They tend to be composed of elements such as grains and domains that are structured hierarchically in a complex manner on several length scales. In materials science, major research efforts have been directed at establishing multiscale models^1^,^2^ that predict the evolution of the entire structure during important materials transformations such as processing or failure. At present, however, rigorous bottom–up approaches are not computationally feasible, and coarse-scale modelling of average properties often fails as heterogeneous events, such as the nucleation of new grains or cracks tend to govern the structural evolution. As a result, state-of-the art models at best systematize generic features of the static structure. Such complicating factors are also pervasive in other fields such as geosciences, where the establishment of multiscale approaches would be equally important in understanding geological processes.

Simultaneous inspection of the structural dynamics inside crystalline materials on multiple length scales and in three dimensions (3D) would therefore be a major step forward. With a 3D multiscale microscope, the interdependence between various structural elements and their driving forces could be studied directly and without spurious effects from artificial boundaries. Validation of material models could then progress from merely comparing their average properties with observations of the critical local events that determine the behaviour of the material.

Such a tool would ideally be based on diffraction, which readily yields information pertinent to both structure (phase, shape and crystallographic orientation of the structural elements) and, by monitoring the lattice strain, the local stress state. Within the last decade, several diffraction-based 3D mapping methods have appeared, however they are not well suited to simultaneous multiscale characterization. Specifically, electron microscopy methods for 3D crystal mapping are either confined to thin foils^3^ or involve serial sectioning^4^,^5^, implying they are inherently destructive. X-ray diffraction tomography methods such as 3D X-ray Diffraction (3DXRD)^6^,^7^,^8^,^9^,^10^ and Diffraction Contrast Tomography (DCT)^11^,^12^ are now capable of mapping of up to 20,000 grains in a sample, but are limited by the detector to a spatial resolution of ~1 μm. 3D methods based on scanning X-ray nano-beams can provide resolutions of 100 nm, but are slow, and the mapped volume is therefore relatively small^13^,^14^,^15^. Furthermore, in these X-ray methods, the overlap of diffraction signals from different volumes in the sample limits the ratio between the average size of the crystalline elements and the sample volume.

In this paper, we propose a strategy for simultaneous multiscale characterization based on a new concept: dark-field X-ray microscopy. It is a full-field imaging technique in which the camera records the image of a layer or the projection of a volume within the sample, enabling fast data acquisition. The technique is therefore highly suited to *in situ* studies of materials and their dynamics, which we demonstrate here by addressing an important topic from metallurgy: the annealing of plastically deformed aluminium.

## Results

### Principle

**Figure 1:**
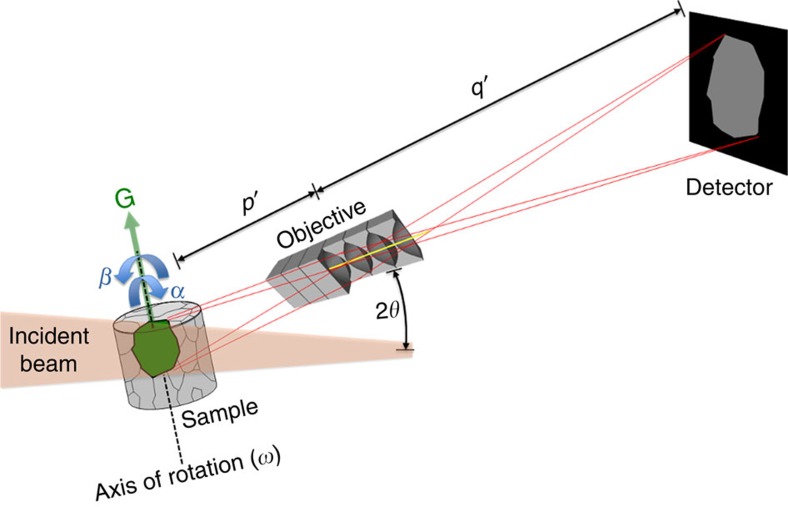
Principle of dark-field X-ray microscopy. A monochromatic beam from a synchrotron source illuminates the sample. An embedded crystalline element (for example, a grain or domain) of choice (green) is aligned such that the beam is diffracted. The objective magnifies the diffracted beam by a factor *M=q′/p′* and generates an inverted 2D projection of the grain. Through repeated exposures during a 360° rotation of the element around an axis parallel to the diffraction vector, G, several 2D projections of the grain are obtained from various angles^30^. A 3D map is then obtained by combining these projections using reconstruction algorithms similar to those developed for CT scanning^32^. If the lattice of the crystalline element exhibits an internal orientation spread, this procedure is repeated for a number of sample tilts, indicated by the angles *α* and *β*. Using a compound refractive lens^18^,^29^ as the objective enables one to enlarge or reduce the spatial resolution and field of view within the sample by varying the number of individual lenses and adjusting *p*′ and *q*′ correspondingly. The diffraction angle 2*θ* is typically 10–30°.

The principle of the dark-field X-ray microscope is shown in Fig. 1. The entire specimen is initially mapped on a coarse scale using 3DXRD or DCT and, if necessary, classical X-ray tomography. This survey facilitates identification of small-scale elements of interest such as grains or domains. Dark-field microscopy then enables us to ‘zoom in’ on these elements and record a magnified high-resolution 3D map. The basic principle of the microscope is that the objective generates a real space image using the diffracted beam as the illumination. A more thorough discussion of dark-field X-ray microscopy in the context of existing X-ray and electron-based microscopy techniques is provided in Supplementary Note 1.

At the finest scale, structural elements often exhibit a near-perfect lattice. A two-dimensional (2D) spatial map of such elements can be acquired in a few seconds, enabling monitoring of the local dynamics. If the element exhibits a minor internal orientation spread, the measurement procedure is repeated while tilting the sample around two perpendicular axes by the angles *α* and *β* in Fig. 1. Furthermore, scanning the scattering angle (labelled 2*θ* in Fig. 1) facilitates 3D mapping of the local stresses through the direct measurement of lattice strain. Details of operation are provided in Methods, while estimates of the spatial and angular resolution and experimental validations of the optical approach are presented in Supplementary Note 2 and Supplementary Fig. 1.

The objective in the dark-field microscope has an inherently small numerical aperture, the implications of which are twofold: first, the angular resolution is superior to conventional transmission electron microscopes (TEMs)^16^. Second, the objective acts as a very effective filter, removing diffraction signals from other diffracting elements within the sample but outside the desired field of view. In favourable circumstances, it then becomes possible to study a specific element out of 10^10^ or more within the illuminated volume—a key requirement for multiscale studies in bulk materials.

Theoretical optics predicts that a spatial resolution of 10 nm is feasible with the dark-field X-ray microscope. For weakly absorbing samples, X-rays of intermediate energies (5–15 keV) may be used in combination with high-quality Fresnel zone plates^17^. Stronger absorbing samples such as those of prime interest to materials science necessitate X-ray energies above 15 keV and the use of compound refractive lenses instead of Fresnel zone plates^18^,^19^,^20^. While the manufacturing technology for CRLs is still maturing, state-of-the-art optics currently yields a spatial resolution of ~100 nm.

### Study of recovery in aluminium

Here, we present an annealing study of commercial pure aluminium, which had been subjected to 10% tensile deformation. This is a prototypical multiscale material comprising grains with an average size of ~80 μm, each subdivided into a 3D structure of subgrains. The boundaries of these subgrains comprise a dense 2D network of dislocations (line defects), while their interior is almost dislocation-free^9^,^21^,^22^,^23^. From this material, a matchstick-shaped sample of dimensions 0.3 × 0.6 × 8 mm^3^ was prepared.

**Figure 2:**
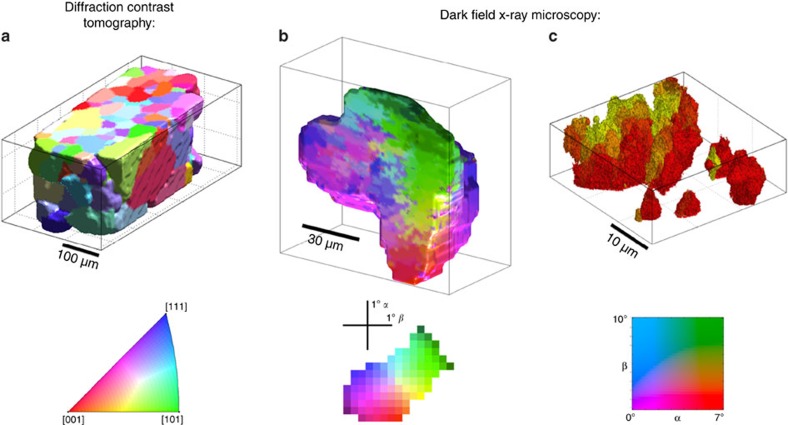
Multiscale mapping of 10% tensile deformed aluminium. Above: (**a**) Part of the X-ray mapping of all grains in the specimen. (**b**) Zooming in on one embedded grain and mapping the intrinsic variation in orientation. A vertical section through the grain is shown for ease of inspection of the spatial heterogeneity. (**c**) Condensing the incoming beam vertically defines a sub-micron layer within the grain. Mapping individual subgrains in 3D is then obtained by ‘stacking’ these layers. The spatial resolution from left to right is 3.5 μm, 1.5 μm and 300 nm, and the angular resolution is 0.5°, 0.15° and 0.03°, respectively. Below: The corresponding keys for the orientation maps. (**a**) the colour scheme is represented in an inverse pole figure. (**b**,**c**) the colour schemes symbolize the required tilts *α* and *β* to align the [200] direction with the diffraction vector.

The grains in this sample were first mapped on a coarse scale with DCT (Fig. 2a), after which we identified an embedded grain with a 200 reflection positioned within a few degrees of the original tensile axis. Subgrains of this orientation are equiaxed with an average size of ~3 μm (ref. 24).

The grain initially selected for microscopy exhibited a total intrinsic orientation variation of 3°. To map the entire grain, the sample was tilted in small steps around two orthogonal axes by the angles *α* and *β* (see Fig. 1). For each tilt position, the sample was rotated about the diffraction vector by 360°, enabling a 3D volume of the grain to be reconstructed in a manner analogous with tomography (c.f. Supplementary Note 3). Combining the maps from different tilts enables a 3D map of local orientations to be reconstructed, shown in Fig. 2b. Here, an intermediate subdivision of the grain into regions on a 10 μm scale is observable. Formation of such orientation domains is attributed to both the instability of homogeneous plastic deformation in the interior of the grain, and the influence of neighbouring grains (evident in the green part of the grain at the top of the figure).

The highest 3D resolution, and thus the final ‘zoom’ state, can be obtained by illuminating individual layers in a grain using an X-ray beam focused to a sub-micron size in the vertical direction only (that is, a line beam), and then stacking these layers into a 3D volume. This approach illuminates a smaller volume of material containing fewer subgrains, allowing individual subgrains to be more clearly visible in the raw dark-field images (see Supplementary Fig. 1). As above, a map of the subgrain orientations within the layer of the sample can be constructed from a series of images at differing tilt angles (*α*, *β*). Then, by acquiring 40 layers of this kind, the subgrains within a 10 × 200 × 200 μm^3^ volume can be mapped with a resolution of ~300 nm. A part of this map is shown in Fig. 2c.

**Figure 3:**
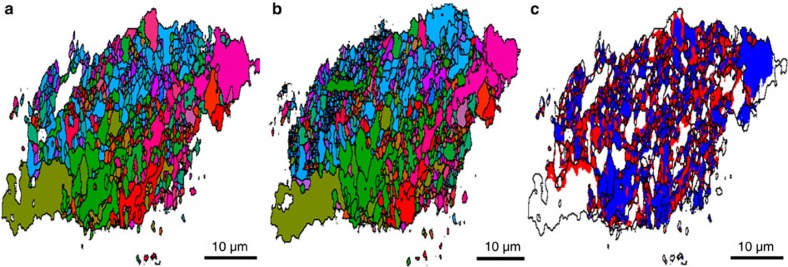
Study of recovery in tensile deformed aluminium. Shown is a 2D map of one layer in the sample, (**a**) after annealing for 0.5 h at 246 °C, and (**b**) after additional annealing for 0.5 h at 257 °C. The colours symbolize the orientation of the [200] direction of the crystalline lattice with respect to the specimen reference system, *cf.* Fig. 1. Shown in (**c**) is a difference map with white, blue and red representing local changes in orientation below 0.01° between 0.01° and 0.2° and above 0.2°, respectively. The spatial resolution is 300 nm.

Tracking subgrain dynamics during the annealing process required sampling a large range of orientations with high spatial precision within a narrow time frame (minutes). As mapping the full volume of the grain in 3D would have been too time-consuming, we instead illuminated only a single layer using the line beam. Proceeding in this manner, a single layer was successively mapped while annealing the sample in steps of increasing temperature. The results for two subsequent temperature steps are shown in Fig. 3a,b. Experimental and analysis details are described in Methods, while an orientation map corresponding to an additional higher temperature is given in Supplementary Fig. 2.

The considerable softening of aluminium that occurs during annealing treatments is of key importance in many processing operations. The structural changes responsible for this effect are collectively known as recovery^25^; however, the underlying physical mechanisms have been difficult to ascertain without access to multiscale experimental methods. In particular, it is not clear whether observations by *in situ* TEM^26^ are influenced by the free surfaces inherent to TEM. Our results, as evidenced from the orientation difference map in Fig. 3c, indicate that the majority of subgrains either exhibit no resolvable rotation (white areas within the grain) or small rotations of <0.2° (blue areas). The remaining red areas represent locations where the orientation has changed >0.2° during annealing. These areas are primarily located adjacent to subgrain boundaries and represent regions, which have been traversed by moving subgrain boundaries. After the passage of the subgrain boundary, these regions adapt their orientation to that of the adjacent subgrain. These findings are consistent with conjectures in the literature of subgrain growth by subgrain boundary motion^25^, but this is the first time the processes have been directly verified and quantified.

## Discussion

These results demonstrate the capability of dark-field X-ray microscopy as a probe for studying the dynamics of a representative ensemble of subgrains during processes such as plastic deformation or recrystallization. More broadly, such studies serve to improve the general understanding of domain evolution in crystalline materials during phase transformations.

Beyond the work presented here, the dark-field approach might also be applied to non-crystalline materials by performing microscopy on small angle X-ray scattering or amorphous scattering signals^27^. Furthermore, with a dedicated and optimized set-up at an upcoming diffraction-limited synchrotron storage ring^28^, we estimate that the data acquisition speed can be improved by several orders of magnitude, making this a promising technique for studying more challenging dynamic processes *in situ* or *in operando*.

In conclusion, we have demonstrated a non-destructive technique for the comprehensive multiscale 3D mapping of crystallographic information. Full orientation mapping may be achieved by acquiring two maps of the kind shown in Fig. 2b, corresponding to two independent diffraction vectors. Furthermore, the large sample-to-objective distance is favourable for *in situ* and *in operando* studies with complex sample environments. In addition to opening a new door to multiscale modelling, the technique is also highly suited to applied studies of functioning devices and components, for example, where the behaviour of selected internal nanoscale grains or domains at interfaces or crack tips governs performance or lifetime.

## Methods

### Microscope configuration

This work was performed at beamline ID06 at the ESRF. A Si(111) Bragg–Bragg double crystal monochromator located 35.8 m from the source selected 17 keV photons (*λ*=0.728 Å) emitted by a cryogenic permanent magnet undulator. A primary condenser was used, comprising a CRL transfocator^29^ located 38.7 m from the source and configured with seven one-dimensional Be lenses with an apex radius of curvature of 200 μm and an effective aperture of 860 μm.

Situated 59 m from the source, the dark-field microscope comprised an optional secondary condenser, a sample stage, a near-field imaging detector, an objective and two far-field detectors of differing field of view and resolution. The objective projects a magnified and inverted image of the sample on one of the two far-field detectors, both located at the downstream end of the hutch at *p*+*q*=4.750 m.

The secondary condenser was used to make an incoming line beam through two configurations. In the first, a Si-based CRL was used, positioned 232 mm from the sample and containing eight one-dimensional lenses with an apex radius of curvature of 6.2 μm, yielding an effective aperture of 40 μm and a divergence of 0.01°. In the second, a Be-based CRL was used, positioned 500 mm from the sample and containing 48 2D Be lenses with an apex radius of curvature of 50 μm, yielding an effective aperture of 277 μm and a divergence of 0.03° (see Supplementary Note 2 for description of the optical calculations performed here). The former configuration is associated with a superior definition of the beam (<1 μm versus 5 μm vertical full width at half maximum), and an improved angular resolution, while the latter provides more flux on the sample.

The sample tower required six degrees of freedom to enable the rotation of the sample around the diffraction vector necessary for 3D mapping with topo–tomography^30^. As with the configuration of Ludwig *et al.*^30^, the base tilt is set to the Bragg angle *θ*, while the two tilt angles *α* and *β*, situated above, are used to align the lattice planes of the selected grain with the axis of the rotation stage, *ω* and thus the diffraction vector G.

The near-field imaging detector consisted of a scintillator screen coupled to a FReLoN CCD camera by microscope optics, giving an effective spatial resolution of 3.5 μm and a field of view of 7 × 7 mm^2^. It was used to align the instrument and sample, to characterize the beam and to perform the DCT mapping.

The objective was a CRL containing 72 2D Be lenses with an apex radius of curvature of 50 μm. In its focused position, the centre of the objective was aligned on the diffracted beam at a distance of 342 mm from the sample. Using the thick lens expressions given in Supplementary Note 2 the focal distance is *f*=244.7 mm, and the magnification *M*=−16.08. The resulting effective aperture becomes *D*_eff_=235 μm and the numerical aperture NA=0.00035.

From these numbers it follows that the angular resolution (full width at half maximum) in directions *β* and *θ* are Δ*β*=0.11° and Δ2*θ*=0.039°, respectively. The corresponding resolution in reciprocal space becomes Δ*q*_radial_=|q_200_|Δ2*θ*/2tan (*θ*)=0.0060 Å^−1^and Δ*q*_azimuth_=|q_200_|Δ*β*=0.0058 Å^−1^.

With our configuration, the angular resolution in *α*, Δ*α*, is dominated by the divergence of the incoming beam. Using only the primary condenser, the resolution in the corresponding direction in reciprocal space is Δ*q*_rock_=|q_200_|Δ*α*~0.0003 Å^−1^. In this configuration the reciprocal space resolution is strongly anisotropic with Δ*q*_rock_≪Δ*q*_radial_≈Δ*q*_azimuth_. The resolution can be made approximately isotropic by ‘rocking’, in which images are acquired while rotating the sample in *α*. The microscope’s 3D field of view in direct space corresponds to a region in reciprocal space with a relative volume of order 10^−9^ in comparison with the Brillouin zone (the Voronoi cell around the origin in reciprocal space).

The far-field detectors were (a) a high-resolution 2D detector comprising a scintillator screen coupled to a FReLoN CCD camera with microscope optics to give a spatial resolution and field of view of 2.5 μm and 3 × 3 mm^2^, respectively, and (b) a low-resolution 2D detector comprising a scintillator screen coupled to a Basler CCD camera with wide-angle optics to give a spatial resolution and field of view of 55 μm and 50 × 50 mm^2^, respectively. The high-resolution FReLoN camera was used for the microscopy images which, accounting for the X-ray magnification of 16.1, gave a resolution and field of view at the sample position of 150 nm and 0.2 × 0.2 mm^2^. However, to improve the signal-to-noise ratio, the detector pixels were binned 2 × 2 to give a final resolution of 300 nm. The large field of view Basler camera served to easily identify and align grains, and to facilitate high-resolution reciprocal space mapping of entire grains to determine their mosaic spread and strain heterogeneity. It may also be used to provide a fast overview of grain and subgrain dynamics in the sample^9^.

### Beamtime details

This work was carried out during three beamtimes related to the 3D imaging and annealing studies illustrated in Fig. 2a–c and Fig. 3, respectively. The optical configurations used in these beamtimes were individually optimized and therefore differed slightly from one another. The reconfiguration between these beamtimes meant that the grains investigated in the three beamtimes were not necessarily the same. The development of a dedicated dark-field X-ray microscope would enable these three measurements in fast succession on a single element of interest.

The first beamtime employed the topo–tomography approach without use of a secondary condenser. The spatial resolution of each image was close to the predicted value of 300 nm. The resolution in 3D, however, was limited by the sphere-of-confusion of the rotation and tilt stages below the sample, estimated to be ~1.5 μm. The grain map shown in Fig. 2b is based on an importance-guided 3D scanning procedure, derived from intensities observed in the pole figure of the reflection. A total of 106 topo–tomography scans were recorded at differing sample tilts *α* and *β*, each comprising 36 projections over a full 360° rotation in *ω*. Each image was integrated over a range of 0.15° in the base tilt angle, *θ*. The scanning procedure sampled a grid of 1.8 × 2.1° in *α* and *β*, respectively, with a step size of 0.15° for both tilts. Due to time constraints, this scan was limited to a sub-region corresponding to 60% of the total area around the most intense (*α*, *β*) position. The acquisition of the map took 9.5 h, of which only 20% corresponded to actual X-ray exposure due to overheads in the scanning procedure.

The second beamtime aimed to demonstrate the high-resolution 3D mapping by utilizing a Si secondary condenser to make an incident line beam with a vertical dimension of <1 μm. The narrow beam illuminates fewer subgrains, and these now become distinct in the raw data as shown in Supplementary Fig. 2a, while the sphere-of-confusion issue is much less pronounced. In this way, a 200 × 200 × 10 μm^3^ volume was mapped layer-by-layer by acquiring 40 layers in steps of 250 nm. Each layer involved a 2D scanning procedure with a range of 0.3° and steps of 0.03° in *α* at a constant value of *β*. Each image was integrated during a three-second exposure over a range of 0.03° in the base tilt angle, *θ*. Having corrected for the finite beam height by a deconvolution procedure, a part of the resulting 3D map is shown in Fig. 2c. This map is not space filling due to the limited orientation range sampled.

For the third beamtime, the Be-based secondary condenser was used, which has a substantially larger numerical aperture and better transmission than the Si lenses used previously. This increased the flux. The incident line beam height is 5 μm and, as such, (possibly overlaying) 2D projections of grains are observed. For the time-resolved study, this set-up proved a good compromise between the image contrast of individual subgrains and counting statistics. Mapping a single layer of interest involved a 2D scanning procedure with a range of 0.8° and steps of 0.01° in *α* and a range of 0.8° and steps of 0.1° in *β*. Each image was integrated during a five second exposure over a range of 0.01° in the base tilt angle, *θ*. An example of raw data is shown in Supplementary Fig. 1b.

The CRL and the far-field detector positions are pre-aligned in the direct beam with the sample removed. When a grain of interest has been identified, the grain is centred and the relevant diffraction vector and the rotation axis are aligned using the near-field camera and a photodiode close to the sample. Next, the grain is rotated to scatter in the direction of the CRL and far-field detector. Finally, the near-field camera is translated out of the diffracted beam and the CRL is subsequently translated into it.

### Sample

A tensile specimen of 1 mm thickness, 12 mm gauge length and 4 mm width was machined from a sheet of commercial aluminium (AA 1050), which had been rolled and annealed to an average grain size of ~80 μm. The specimen was deformed in tension to 10% and exhibited only a weak crystallographic texture^31^. From the tensile specimen, a needle-shaped sample was cut and ground to its final dimensions of 0.3 × 0.6 × 8 mm^3^, with its long direction parallel to the tensile axis.

The sample was subsequently placed in the dark-field X-ray microscope with the tensile axis parallel to the axis of rotation. The illuminated volume of the sample (0.3 mm in height) during the first beamtime comprised 283 grains, identified by the DCT analysis.

### The annealing study

To heat the sample *in situ*, a hot air blower was placed ~3 mm from the sample. The true temperature at the position of the grain was determined by measuring the angular change of the (200) reflection during heating, as observed on the large field-of-view far-field 2D detector. On the basis of the known linear expansion coefficient for pure aluminium, the change in *d*-spacing and temperature were inferred. The accuracy in temperature determined in this way is estimated to be better than 1 K.

The sample was annealed by progressively increasing the temperature in steps until recrystallization took place. At each step, the temperature remained constant for 0.5 h (annealing temperature), followed by reducing the temperature by 30 K (measuring temperature) and maintaining this temperature for 1.5 h during the measurements. The purpose of this 30 K temperature reduction was to arrest the structural change during the measurement periods. Our data pertains to three steps in the vicinity of the annealing temperature at 246, 257 and 268 °C.

Care was provided in terms of ensuring thermal stability. During each of the three 1.5-h measurement intervals, the temperature stability determined from the Basler detector data was of the order of 0.1°. To verify that the same layer was monitored at the various temperature steps, high-resolution X-ray radiography was performed on markers on the surface of the sample. As a result, we found that the layers mapped at lower temperatures are remarkably similar.

The high-resolution dark-field microscopy images were post-processed in several steps: (1) the background was removed by subtracting an image of the detector dark current and (2) ‘hot’ pixels were removed by interpolation. (3) The images were recombined into four-dimensional (*x*, *y*, *α*, *β*) space and (4) connected components with four-dimensional coordination were identified. The coordination range reflected the differing angular resolution in *α* and *β* as determined by the numerical apertures of the condenser and objective. (5) Each component was assigned an average orientation corresponding to its centroid position in (*α*, *β*). This process identified ~1,800 components at each temperature, with a size distribution skewed towards small components of five or less voxels, reflecting findings by TEM that well-identified subgrains at this level of deformation tend to be surrounded by even smaller dislocation-free units in the boundary regions surrounding the subgrains (the notion of subgrains for the connected components becoming therefore somehow ambiguous). Notably, the size distributions of the components identified at the three temperatures were almost identical. Hence, on average, little growth took place in the measured temperature regime; nevertheless, some subgrain boundary motion seem to occur.

To provide better insight in the subgrain dynamics and to ease visualization, we generated 2D maps by defining the orientation for each (*x*, *y*) position as the (*α*, *β*) values that maximized the measured intensity. In this way, the maps presented in Fig. 3a,b (main article) and Supplementary Fig. 2a,b were generated. Connected components in these maps are defined as subgrains; the boundaries between them are marked with a black outline and reflect orientation differences above the resolution limit of 0.01°. The orientation difference maps in Fig. 3c and Supplementary Fig. 2c are constructed by comparing orientations in the two maps pixel by pixel. The subgrain boundaries associated with the lower temperature are overlaid for reference purposes.

By inspection of Fig. 3c, we find that most subgrains can be traced from one temperature to the other as the local subgrain topology changes little during annealing. However, a sizeable area fraction undergoes a reorientation by an amount that is larger than the instrumental resolution of 0.01°. A histogram of the local reorientation angles is shown in Supplementary Fig. 3. On the basis of this result, a level of 0.2° is used to distinguish between ‘low-angle’ rotation and ‘high-angle’ rotation in Fig. 3c. We find that the high-angle part (shown in red) mainly appears in connection with small subgrains and near the interface between subgrains. Such reorientations are, therefore, seen as being consistent with the movement of a subgrain boundary, which changes the local orientation of a pixel to that of its neighbour.

Supplementary Figure 2 conveys the advances in the structural evolution during further annealing for 0.5 h at 268 °C. Similar to the previous annealing step, individual subgrains can still be traced when comparing the maps before (Supplementary Fig. 2a) and after (Supplementary Fig. 2b) the annealing step. The orientation difference map highlights the same features with some of the area showing no (white) or small reorientations below 0.2° (blue) and larger reorientations (red) mainly located in the vicinity of subgrain boundaries. As seen from the histogram in Supplementary Fig. 3, both the area undergoing reorientation and the reorientation angles during this step are larger than during the previous annealing step (0.5 h at 257 °C). This may indicate a progress in the recovery due to the temperature increase, but could also be caused by increasing difficulties in the alignment of the sample during heating.

During the annealing process studied here, the small rotations of entire subgrains are consistent with the small orientation changes associated with the climb and annihilation of dislocations, while the larger rotations in the vicinity of the subgrain boundaries indicate subgrain boundary motion. This is the first time that such effects are quantified directly. More generally, this study demonstrates how dark-field microscopy can be varied in terms of both direct space and reciprocal space resolution to optimize the statistics of structural units.

## Author contributions

H.S., A.K. and W.L. contributed equally—they developed most of the instrumentation and performed the data analysis. C.D. was in charge of the instrumentation programme at the beamline. W.P. provided the samples and expertise on recovery; S.S. was in charge of algorithms, I.S. of sample preparation and A.S. of CRL optics. H.F.P. conceived the idea of dark-field microscopy and was responsible overall.

## Additional information

**How to cite this article:** Simons, H. *et al.* Dark-field X-ray microscopy for multiscale structural characterization. *Nat. Commun.* 6:6098 doi: 10.1038/ncomms7098 (2015).

## Supplementary Material

Supplementary Information Supplementary Figures 1-3, Supplementary Notes 1-3 and Supplementary References.
