# Supplementary material for: Dark-field X-ray microscopy for multiscale structural characterization
Source: Nat Commun. 2015 Jan 14;6:6098. doi: 10.1038/ncomms7098 (PMC4354092; doi:10.1038/ncomms7098)
Supplement: Supplementary Information — Supplementary Figures 1-3, Supplementary Notes 1-3 and Supplementary References. [file ncomms7098-s1.pdf]

Supplementary Figures

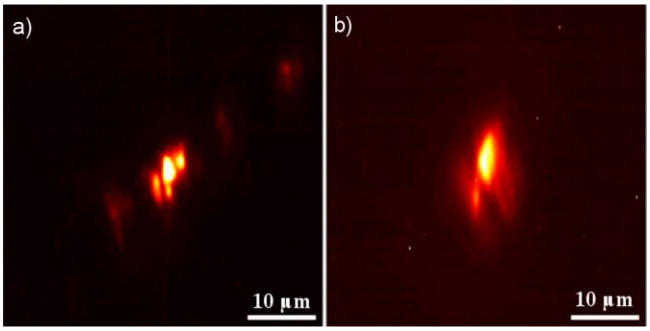

Supplementary Figure 1: Raw data. These are measured at a single orientation of the sample showing intensity contributions from individual subgrains: a) the second beamtime, which used a Si condenser to produce a thinner line beam with lower angular convergence (0.01°) and thus demonstrated higher angular resolution evidenced in clear and well-defined regions of intensity, and b) the third beamtime, which utilised Be lenses for a larger, more convergent (0.03°) line beam to illuminate more subgrains with higher intensity but lower angular resolution, evidenced by the more diffuse intensity. The scale bar is 10 µm.

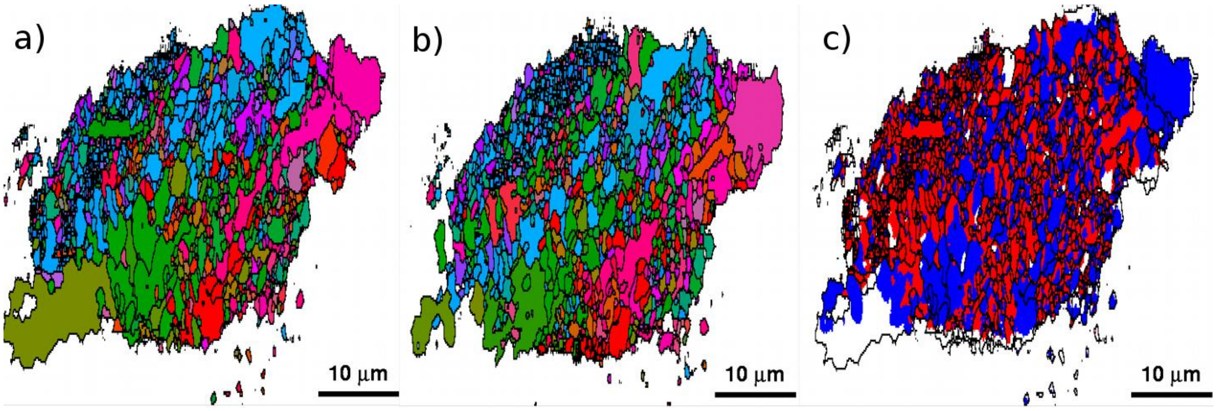

Supplementary Figure 2: Data for the second temperature step. This figure is plotted analogously to Fig 3. Shown is a) the 2D map representing 257 ºC (copy of Fig 3b), b) a map of the same layer after further annealing for 0.5 h at 268 ºC, and c) the orientation difference map between these two temperature steps.

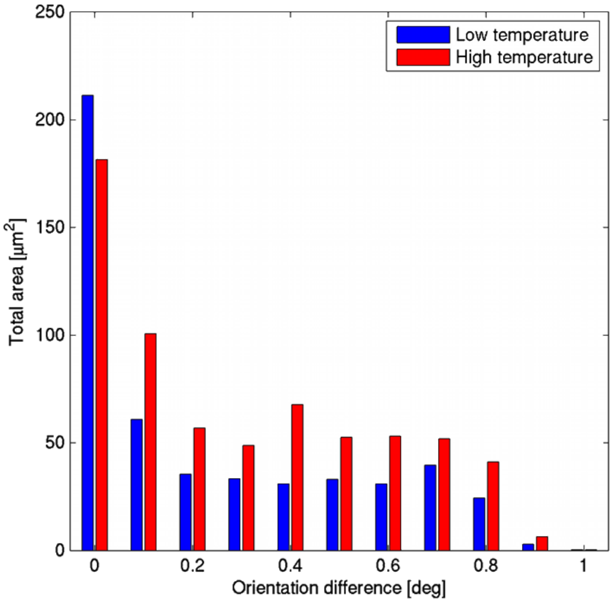

Supplementary Figure 3: Histogram of local reorientation angles. Shown are those associated with the low and high temperature annealing steps (246-257˚C and 257-268˚C, respectively).

Supplementary Note 1

Recent years have seen significant progress in both full-field and scanning x-ray microscopy techniques[1,2]. In full-field imaging, the entire sample is typically illuminated. Two significant examples are bright field x-ray microscopy[3,4], a technique often used for biological and environmental samples where the direct beam is used for phase contrast imaging at x-ray energies up to 15 keV, and high-resolution x-ray microscopy[5], which combines phase contrast and small angle diffraction modalities into a powerful tool for the *in situ* study of soft-matter and colloidal systems such as self-organizing dispersions[6]. Scanning x-ray microscopy, on the other hand, utilizes nanometer-size x-ray beams, and creates a 3D image by scanning the sample with respect to this beam. Such methods are highly versatile and enable 3D mapping with a resolution of 10 nm using coherence-based methods[7,8,9]. Dark field imaging through grating interferometry is another powerful emerging of providing local information using x-rays[10] or neutrons[11]. Using the small angle scattering information it probes the average nanoscale structure, albeit with spatial resolution of a few micrometers.

The aforementioned techniques are all difficult to adapt to multiscale direct-space mapping studies. In contrast, dark field x-ray microscopy exploits wide-angle diffraction contrast for full field multiscale studies of bulk crystalline materials of mm size. The concept of dark field microscopy is known from electron microscopy[12], where several significant developments have occurred. For

example, Liu *et al.* recently demonstrated a TEM method for the 3D mapping of nano-crystalline domains with spatial resolution of 1 nm by conically scanning the incoming beam[13]. However, the samples that can be studied in this manner are thin foils and the method does not support stress characterisation.

A key characteristic of dark field x-ray microscopy is the small numerical aperture *NA* of the objective which, in our case $NA = 0.00035$ (as calculated below). Although this restricts spatial resolution[14] to $d = 0.61\lambda/NA$ (110 nm in our case), this restriction was insignificant in comparison to the optical aberrations and blurring arising from the vibrations and mechanical errors inherent in the *ad hoc* setup used. Nonetheless, a small numerical aperture has two significant advantages:

1. The angular resolution is high in all three directions (α, β, 2θ). Specifically, the resolution in α, $\Delta\alpha$, is defined by the divergence of the condensed incident beam or, when a condenser is not used, the Darwin width of the crystal[15], while the resolution in β and 2θ are both defined by the numerical aperture according to $\Delta\beta = 2NA/sin(2\theta)$ and $\Delta 2\theta = 2NA$. Furthermore, the angular resolution and mapping rate can be manipulated by rotating α or β during exposures, enabling zooming in reciprocal space.
2. The objective is an effective filter in reciprocal space, acting as a collimator for stray radiation from outside the intended field-of-view. In this optical configuration, the probability that an arbitrarily oriented element scatters within the reciprocal space sampled by the microscope is 10^{-8} to 10^{-9} (see below). Combined with the ability of 3D reconstruction algorithms to filter such stray radiation analogously to local tomography[16,17], we argue that dark field x-ray microscopy can map individual structural elements despite the x-ray beam illuminating $\approx 10^{10}$ or more.

A limitation of dark field x-ray microscopy is that mapping individual structural elements requires prior knowledge of their orientation within the sample. For example, it was known that the orientation spread of the deformed aluminium grain presented in Fig. 2b was 3°. Such information can be readily obtained either by exploiting known orientation relationships (e.g. between parent and child grains after phase transformations) or, more commonly, by initially mapping the structure on a coarse scale, and using this information to identify elements of interest for mapping in high resolution (see Fig. 2).

Mapping axial strain is facilitated by a combined θ-2θ scan. Here, θ is the base tilt at the bottom of the sample tower, while the change in 2θ is achieved by a combined rotation and translation of the objective and the far field detector. Such a measurement of a strain-free material will result in the line profile through 2θ for any point in the image plane, which is approximately Gaussian with a

width Δ2θ corresponding to the numerical aperture. For the work presented here, the strain profile $\Delta d/d = -\Delta\theta\ cot(\theta)$ will have a full-width-at-half-maximum (FWHM) of about 1.8 10^{-3}. In cases where the sample is illuminated by a line-beam, the axial strain is defined by the centre-of-mass of this profile. Notably, the relative width and shape of the line profile are similar to those encountered in neutron strain scanning[18], implying the strain resolution for dark field microscopy will be similar (*i.e.* 10^{-4}).

Supplementary Note 2

The thin lens approximation is not adequate to calculate the optical parameters for a CRL-based condenser or objective[19], instead necessitating an approach accounting for the thickness of the lens stack. For general reference and use in the following we utilise a ray transfer matrix formalism[20] providing analytical expressions. Introducing the number of lenses, N, the lens thickness, T, the reduced distances $p' = p - NT/2$, $q' = q - NT/2$ and the normalised lens thickness $\Lambda = NT/f_0$, the corrected thin lens formula becomes:

$$\frac{1}{p'} + \frac{1}{q'} = \frac{\tan\sqrt{\Lambda}}{f_0\sqrt{\Lambda}} + \frac{f_0\sqrt{\Lambda}\tan\sqrt{\Lambda}}{p'q'} \qquad (1)$$

Here, $f_0 = R/(2\delta N)$ is the focal distance in the thin lens approximation, with δ being the refractive index decrement ($n = 1-\delta+i\beta$), and R the radius of curvature. The following expressions hold for the thick lens focal distance, f, and magnification, M:

$$f = f_0\sqrt{\Lambda}\cot\sqrt{\Lambda} \qquad (2)$$

$$M = \frac{q'}{p'}\cos\sqrt{\Lambda} - \frac{f_0\sqrt{\Lambda}}{p'}\sin\sqrt{\Lambda} \qquad (3)$$

The finite thickness of the lens also implies a correction to the numerical aperture, due to both absorption and because the rays travel in sinusoidal path within the lens[19]. Treating the former effect first, the resulting effective aperture D_{eff} is[21]

$$D_{eff} = 2R_0\sqrt{[1 - \exp(-a_p)]/a_p} \qquad (4)$$

Here, $2R_0$ is the physical aperture of the lens, $a_p = \mu NT/2$, and μ is the linear absorption coefficient. The latter effect is expressed in terms of the relevant lens diameter $D=2R_0$ by[20] :

$$NA = \frac{D}{2p'\sqrt{1+f_0^2\Lambda/p'^2}} \qquad (5)$$

Supplementary Note 3

As mentioned, the experiments reported were performed using an *ad hoc* set up. In perspective, sample goniometers with the relevant degrees of freedom exist with spheres-of-confusion below 50 nm. Another critical source of error is the stability of the sample position with respect to the objective, however, this can be reduced drastically through increased mechanical stiffness. Furthermore, being a full field imaging method, dark field x-ray microscopy provides the possibility of compensating for parasitic movements in data processing through the use of image registration.

Supplementary References

1. Schroer, C.G., *et al.* Hard X-ray full field microscopy and magnifying microtomography using compound refractive lenses. *Nucl. Instr. Methods Phys. Res. A* **467–468** , 966–969 (2001).

2. Sakdinawit, A., Attwood, D. Nanoscale X-ray imaging. *Nature Photon.* **4**, 840–848 (2010).

3. Weilun C., Harteneck, B.D., Alexander Liddle, J., Anderson, E.H. & Attwood, D.T. Soft X-ray microscopy at a spatial resolution better than 15 nm. *Nature* **435**, 1210-1213 (2005).

4. Andrews, J.C., *et al.*, A high resolution, hard x-ray bio-imaging facility at SSRL. *Synchrotron Radiat. News*. **21**, 17–26 (2008).

5. Bosak, A., Snigireva, I., Napolskii, K.S. & Snigirev, A. High-resolution transmission x-ray micrcoscopy: A new tool for mesoscopic materials. *Adv. Mat.*, **22**, 3256, (2010).

6. Byelov, D. *et al.* In situ hard X-ray microscopy of self-assembly in colloidal suspensions. *RSC Advances*, **3**, 15670-15677 (2013).

7. Rodenburg, J.M., *et al.* Hard-X-Ray Lensless Imaging of Extended Objects, *Phys. Rev. Lett.* **98**, 034801 (2007).

8. Robinson, I. & Harder, R. Coherent X-ray diffraction imaging of strain at the nanoscale. *Nat. Mater.* **8**, 291 - 298 (2009).

9. Chapman, H.N., *et al*. High-resolution *ab initio* three-dimensional x-ray diffraction microscopy. *J. Opt. Soc. Am. A.* **23**, 1179-1200 (2006).

10. Pfeiffer, F., Bech, M., Bunk, O., Kraft, P., Eikenberry, E.F., Brönnimann, Ch., Grünzweig, C. & David, C. Hard-X-ray dark-field imaging using a grating interferometer. *Nat. Mater.* **7**, 134 -137 (2008).

11. Strobl, M., Grünzweig, C., Hilger, A., Manke, I., Kardjilov, N., David, C. & Pfeiffer, F. Neutron Dark-Field Tomography. *Phys. Rev. Lett.* **101**, 123902 (2008).

12. Williams, D.B. & Carter, C.B. Transmission Electron Microscopy (Springer, 2009).

13. Liu, H.H., *et al.* Three-dimensional orientation mapping in the transmission electron microscope. *Science* **332**, 833-834 (2011).

14. Born, M. & Wolf, E. Principles of optics (Cambridge,1999).

15. Als-Nielsen, J. & McMorrow, D. Elements of Modern X-ray Physics (Wiley, 2011).

16. Faridani A., Ritman, E.L., Smith, K.T. Local tomography. SIAM *J Appl. Math.* **52**, 459-484 (1992).

17. Kyrieleis, A., Titarenko, V., Ibison, M., Connolley & Withers P.J. Region-of-interest tomography using filtered backprojection: assessing the practical limits. *J. Microsc.* **241**, 69-82 (2011).

18. Santisteban, J. R., Daymond, M. R. James, J. A. & Edwards, L. ENGIN-X: a third-generation neutron strain scanner. *J. Appl. Cryst.* **39**, 812–825 (2006).

19. Kohn, V.G. An Exact Theory of Imaging with a Parabolic Continuously Refractive X-ray Lens. *JETP* **97**, 204–15 (2003).

20. Poulsen, S.O. & Poulsen, H.F. Efficient Analytical Approaches to the Optics of Compound Refractive Lenses for Use with Synchrotron X-rays. *Metall. Mater. Trans A.* (2014). In print.
